# Compensatory mutations modulate the competitiveness and dynamics of plasmid-mediated colistin resistance in *Escherichia coli* clones

**DOI:** 10.1038/s41396-019-0578-6

**Published:** 2020-01-02

**Authors:** Qiu E. Yang, Craig MacLean, Andrei Papkou, Manon Pritchard, Lydia Powell, David Thomas, Diego O. Andrey, Mei Li, Brad Spiller, Wang Yang, Timothy R. Walsh

**Affiliations:** 1 Department of Medical Microbiology and Infectious Disease, Division of Infection and Immunity, Cardiff University, Cardiff, CF14 4XN UK; 2 Department of Zoology, University of Oxford, South Parks Road, Oxford, OX1 3PS UK; 3 Advanced Therapies Group, Oral and Biomedical Sciences, School of Dentistry, College of Biomedical and Life Sciences, Cardiff University, Heath Park, Cardiff, UK; 4 Service of Infectious Diseases, Geneva University Hospitals and Faculty of Medicine, Geneva, Switzerland; 5 Beijing Advanced Innovation Centre for Food Nutrition and Human Health, College of Veterinary Medicine, China Agricultural University, 100193 Beijing, China

**Keywords:** Molecular evolution, Antibiotics

## Abstract

The emergence of mobile colistin resistance (*mcr*) threatens to undermine the clinical efficacy of the last antibiotic that can be used to treat serious infections caused by Gram-negative pathogens. Here we measure the fitness cost of a newly discovered MCR-3 using in vitro growth and competition assays. *mcr-3* expression confers a lower fitness cost than *mcr-1*, as determined by competitive ability and cell viability. Consistent with these findings, plasmids carrying *mcr-3* have higher stability than *mcr-1* plasmids across a range of *Escherichia coli* strains. Crucially, *mcr-3* plasmids can stably persist, even in the absence of colistin. Recent compensatory evolution has helped to offset the cost of *mcr-3* expression, as demonstrated by the high fitness of *mcr-3.5* as opposed to *mcr-3.1*. Reconstructing all of the possible evolutionary trajectories from *mcr-3.1* to *mcr-3.5* reveals a complex fitness landscape shaped by negative epistasis between compensatory and neutral mutations. Our findings highlight the importance of fitness costs and compensatory evolution in driving the dynamics and stability of mobile colistin resistance in bacterial populations, and they highlight the need to understand how processes (other than colistin use) impact *mcr* dynamics.

The first discovery of plasmid-borne *mcr-1* gene encoding colistin resistance in 2015 [1], has raised major international concerns for human health as their dissemination within and between species can rapidly confer wide-spread resistance to colistin, a last-resort antibiotic deployed against carbapenem-resistant pathogens. Antibiotic resistance is usually associated with a fitness cost [2, 3], and this cost helps to limit the spread and maintenance of resistance, for example by reducing the ability of bacteria to transmit between hosts [2, 4, 5]. In this case, the expression of *mcr-1* in *Escherichia coli* confers a fitness cost, virulence loss and changes in innate immune response [6]. These changes suggest that restricting colistin use, for example by banning the use of colistin as a growth promoter, could lead to the decline of *mcr-1*.

A new transferable *mcr-3.1* gene with only 45.0% nucleotide homology to *mcr-1*, was firstly identified on an IncHI2-type plasmid in an *E. coli* isolate from pig faeces from China in June 2017 [7]. Two variants of *mcr-3* (*mcr 3.1 and mcr 3.5*) are now highly prevalent in South East Asia (unpublished data), but despite their clinical importance, the impact of *mcr-3* expression on bacterial fitness is still unknown. To test the impact of *mcr-3* expression on bacterial fitness, we cloned *mcr-3* into an inducible expression vector using a strategy as previously described to measure the cost of *mcr-1* [8] (details of strains and plasmids are listed in Supplementary Table 1). Consistent with our previous work, induction of *mcr* expression slowed the growth of *E. coli* Top10 relative to uninduced controls, demonstrating a fitness cost by *mcr* expression (Fig. 1a). Both *mcr-3* variants imposed a smaller fitness cost than *mcr-1*, but the expression of *mcr-3.5* was less costly than that of *mcr-3.1*. We then assessed the effect of MCR-3 on cell viability via LIVE/DEAD® staining with confocal laser scanning microscopy imaging (details see in Supplementary File 1). A minimal reduction in bacterial viability was observed in *E. coli* TOP10 (*mcr-3.5*/pBAD), compared with that of *E. coli* TOP10 (*mcr-3.1*/pBAD) (~3 vs 9%, *p* = 0.0002, see Supplementary Fig.1 and Supplementary Table 4). Furthermore, we investigated whether expression of MCR-3.1 and MCR-3.5 affects bacterial morphology by using transmission electron microscopy. The control strain, *E. coli* TOP10 (pBAD alone), showed normal cellular characteristics with a multi-layered cell outer membrane and cytoplasmic granular density (Supplementary Fig. 2). However, a demonstrable loss of electron density and significant impaired cell wall integrity were observed in both MCR-3.1- and MCR-3.5*-*producing *E. coli* TOP10 cells (Supplementary Fig. 2), which is consistent with our previous findings with *mcr-1* expression [8].

**Fig. 1:**
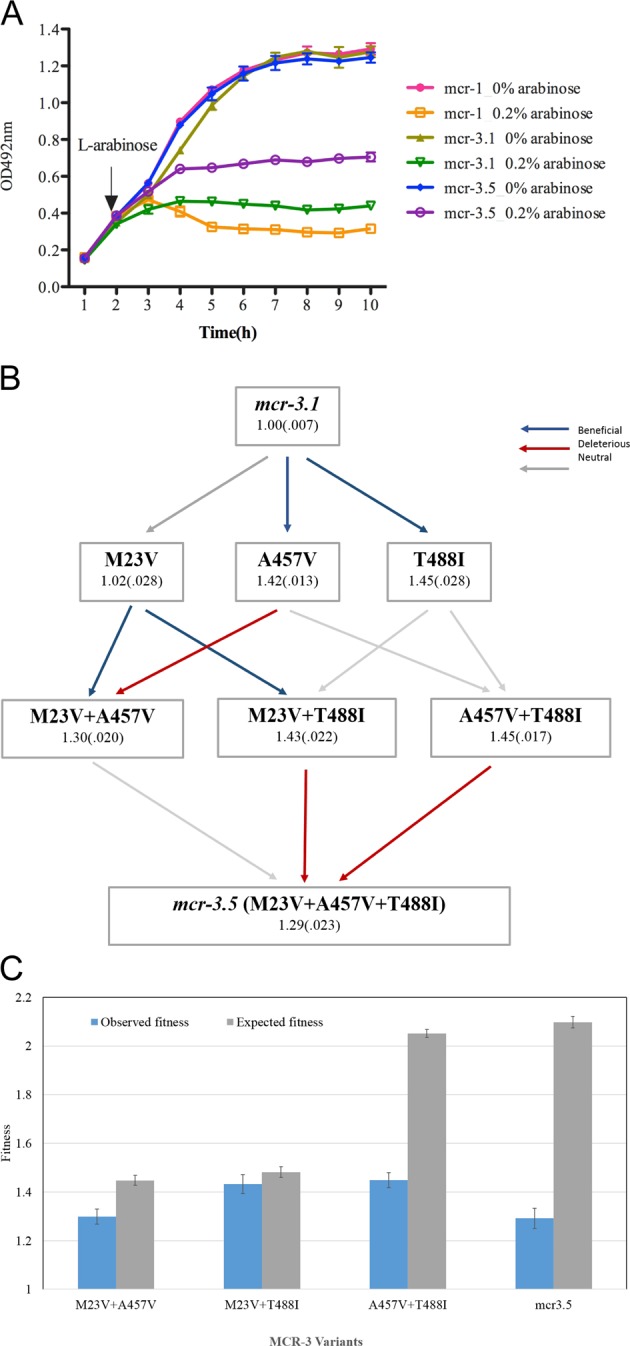
The competitiveness and fitness landscape of *mcr-3* variants. **a** Effects of the expression of three *mcr*-variants, *mcr-1*, *mcr-3.1* and *mcr-3.5* on bacterial growth rate in vitro. The expression of *mcr-* genes was induced by 0.2% (w/v) L-arabinose and bacterial density was measured by microplate reader at every 1 h. The data represent the mean and SD (*n* = 3). **b** The adaptive landscape of colistin resistance *mcr-3.5* conferred by three mutations in *mcr-3.1* gene. Each node displays the amino acid substitution (M23V, A457V and T488I) and its average fitness. This figure shows the fitness landscape connecting MCR3.1 to MCR3.5. Possible evolutionary trajectories are shown with arrows and the fitness of each genotype is given followed by the standard error of fitness. We tested the fitness effect of each mutation using a *t*-test followed by a Bonferroni correction for multiple (*n* = 12 tests). Blue and red arrows show mutations that significantly increase or decrease fitness, respectively, and grey arrows show neutral mutations that do not alter fitness (details in Supplementary Table 6). **c** Epistatic interactions among MCR-3 mutants: this figure shows the observed (blue) and expected (grey) fitness of MCR mutants containing at least two substitutions (+/− S.E). Expected fitness values were calculated using a multiplicative model of fitness and we used the method of propagation of errors to determine the error in expected fitness estimates (Supplementary Table 7).

It is well-established that compensatory mutations can mitigate the fitness cost associated with the expression of antibiotic resistance genes, and therefore allowing resistance genes to be stably maintained in bacterial populations [2]. *mcr-3.1* and *3.5* differ from each other by only three amino-acid substitutions (M23V, A457G, T488I), suggesting that recent compensatory evolution has mitigated the cost of *mcr3* expression. To further understand the compensatory evolution in MCR-3, we reconstructed all of the possible variations between MCR-3.1 and MCR-3.5 (primers and methods shown in Supplementary Tables 2 and 3). Substitutions A457V and T488I had strong compensatory effects when they were inserted into MCR-3.1, increasing fitness by up to 45% (Fig. 1b). However, the double substitution of A457V and T488I did not demonstrate a higher fitness than either of the single mutants, demonstrating negative epistasis between these substitutions (Supplementary Table 7). The remaining substitution (M23V) is either neutral or mildly deleterious. These epistatic interactions generate a complex fitness landscape with two isolated peaks with fitness rates of ~1.3 and 1.45, respectively (Fig. 1b,c). Crucially, only 1 of the 6 possible evolutionary trajectories linking MCR-3.1 to MCR-3.5, gives monotonically increasing fitness (i.e. no deleterious mutation) and this trajectory involves a combination of neutral mutations (M23V, T488I) and only a single compensatory mutation (A457V) (Fig. 1b and Supplementary Tables 5–7). These data clearly show that compensatory mutations can mitigate the cost of MCR-3 expression, but they also highlight the role that epistasis plays in the evolution of MCR enzymes. A key challenge for future work will be to understand how these substitutions alter the substrate binding and/or catalytic activity of MCR-3.

Given that MCR-3 imposes a lower fitness cost than MCR-1, then it follows that MCR-3 should have a higher stability than MCR-1. To test this hypothesis, we passaged cultures of three wild-type *E. coli* strains that harbour both MCR-3 and MCR-1 plasmids over 14 days in the presence and absence of colistin (see details in Supplementary File 1). The dynamics of individual plasmid/host strain combinations were complex, but the ratio of *mcr*-3/*mcr*-1 plasmids consistently increased over time in all three strains, consistent with the low cost of MCR-3 carriage (Fig. 2a and Supplementary Tables 8–12). The dynamics of plasmid populations are complex, but the key difference appears to be that *mcr-1* plasmids were slowly lost, whereas *mcr-3* plasmid populations recovered after an initial period of decline. Interestingly, *mcr*-3 plasmids were able to increase in frequency in the absence of colistin exposure.

**Fig. 2:**
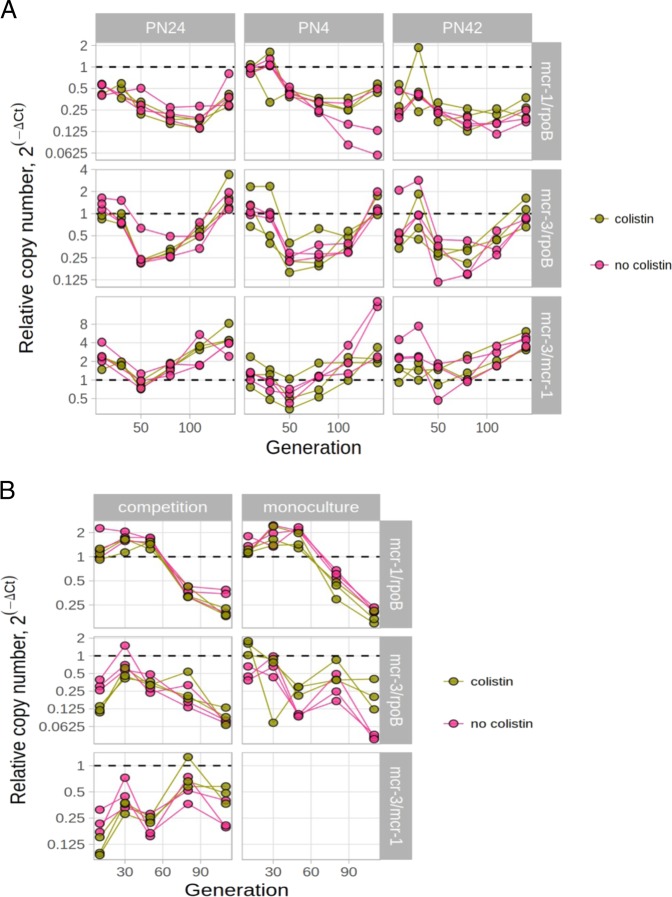
The abundance of *mcr-1* and *mcr-3* plasmids in two competition models. **a** The dynamic changes of *mcr-1* and *mcr-3* plasmids’ copy numbers in wild-type strains. To model the change in *mcr-1* or *mcr-3* copy number across time, we used polynomial regression. The difference in threshold cycle (Δ*Ct*) between either *mcr-1* or *mcr-3* and chromosomally encoded gene *rpoB* were used to calculate their relative copy numbers over time (see Methods in Supplementary File 1). In addition, the difference in threshold cycle (Δ*Ct*) between *mcr-3* or *mcr-1*, which is equivalent to log_2_ of relative copy number, were used as a response variable. In this figure, four fixed variables and their interactions were used as predictors: Strain (PN42, PN4 or PN24), genes (*mcr-1* and *mcr-3*), generations and Colistin (colistin presence/absence). Each strain included three independent replicates which were measured repeatedly over the course of the experiment. For a full model incorporating the effect of host strain, particular gene and presence of colistin, see Supplementary Tables 9–13. The analysis was performed using R (version 3.5.1) and packages lme4 (version 1.1–17) and lmerTest (version 3.0-1). **b** The dynamic changes of *mcr-1* and *mcr-3* genes/plasmids in *E. coli* J53 strain. We used threshold cycle values (*C*_*T*_) measured by qPCR in order to estimate relative copy number (see Methods). Two fixed variables and their interactions were used as predictors in this figure: cultures (monoculture vs mixed cultures), genes (*mcr-1* and *mcr-3*), generations and Colistin (colistin presence/absence). For a full model incorporating the effect of culture, particular gene and presence of colistin, see Supplementary Tables 13–15. The analysis was performed using R (version 3.5.1) and packages lme4 (version 1.1-17) and lmerTest (version 3.0-1).

To further test the effect of MCR production on plasmid stability, we generated tranconjugants of *E. coli* J53 carrying either an *mcr-1* plasmid or an *mcr-3* plasmid. The abundance of both the *mcr-1* and *mcr-3* plasmids declined in transconjugant monocultures, demonstrating that these plasmids impose a fitness cost (Fig. 2b and Supplementary Tables 13–15). Plasmid frequency also decreased over time when the transconjugants were co-cultured. However, the *mcr-3* plasmid had a higher stability than the *mcr-1* plasmid under direct competition, which is consistent with a reduced fitness cost of MCR-3. Our results suggest that the long-term persistence of resistance is influenced by both the fitness effects (e.g. *mcr-3*-positive plasmids carry a smaller cost than that of *mcr-1-* plasmids, see in Fig. 1), and plasmid dynamics (e.g. recovery of *mcr-3*-positive plasmids in spite of costs of gene, see in Fig. 2). Interestingly, the short-term dynamics of the resistance genes can be largely understood in terms of fitness effects, but plasmid dynamics become more important over the longer term.

There is little doubt that the use of colistin is a key driver for the emergence of colistin resistance. Large quantities of colistin have been used in food-producing animals for metaphylaxis or as growth promoters [1, 9, 10], and colistin is still considered a vital antibiotic for treating serious Gram-negative infections [6]. Interestingly, we found that the addition of low concentrations (2 mg/l) of colistin had little or no impact on the outcome of *mcr-1* and *mcr-3* plasmid dynamics (Supplementary Tables 8–15), suggesting that colistin is not the only factor influencing the spread and maintenance of *mcr*-carrying plasmids. Both competition models showed that both the host genotype and gene/plasmid characteristics can influence the long-term co-existence of *mcr* genes.

Understanding the drivers of stability of resistance genes in complex microbial communities is of great importance for predicting the fate of resistance genes and success of bacterial clones in response to ecological challenges (e.g. antibiotics). It is generally understood that the acquisition of antibiotic resistance genes in bacterial communities are deleterious, due to their fitness burden that limiting the spread of resistance genes in the bacterial populations [2, 3, 11]. However, bacteria can and adapt and evolve rapidly to persist in these changing environments through compensatory mutations [11, 12]. In this study, we provided evidence that the compensatory mutations found in *mcr-3* can mitigate the fitness cost imposed by plasmid acquisition and significantly improve the retention of *mcr-3*-positive plasmids. Moreover, although the fitness burden cost occurred in the first 50 generations, both *mcr-1* and *mcr-3* genes can persist in mixed populations, and the relative abundance of *mcr-3*-carrying plasmids increased over time, suggesting that the burden cost are alleviated through compensatory adaptations. In particular, associations exist between resistance genes and bacterial communities via vertical and horizontal transfers, and as *mcr-1* and *mcr-3* plasmids are highly transferable (conjugation rates are as high as 10^−4^, unpublished data), there is the potential risk of *mcr-1* and *mcr-3* genes being rapidly disseminated throughout bacterial communities. In conclusion, our study reveals that (i) both MCR-1 and MCR-3 genes produce fitness costs during bacterial competition; (ii) the costs can be alleviated through compensatory mutations; (iii) both fitness costs and plasmid dynamics play an important role in the persistence of resistance genes within bacterial communities; (iv) our data also highlight the importance of the genetic context of resistance genes/plasmids for understanding the long-term dynamics of resistance.

## Supplementary information

Supplemental Material

## References

[1] Liu Yi-Yun, Wang Yang, Walsh Timothy R, Yi Ling-Xian, Zhang Rong, Spencer James, Doi Yohei, Tian Guobao, Dong Baolei, Huang Xianhui, Yu Lin-Feng, Gu Danxia, Ren Hongwei, Chen Xiaojie, Lv Luchao, He Dandan, Zhou Hongwei, Liang Zisen, Liu Jian-Hua, Shen Jianzhong (2016). Emergence of plasmid-mediated colistin resistance mechanism MCR-1 in animals and human beings in China: a microbiological and molecular biological study. The Lancet Infectious Diseases.

[2] Andersson DI, Hughes D (2010). Antibiotic resistance and its cost: is it possible to reverse resistance?. Nat Rev Microbiol.

[3] Vogwill T, MacLean RC (2015). The genetic basis of the fitness costs of antimicrobial resistance: a meta-analysis approach. Evolut Appl.

[4] Bell G, MacLean C (2018). The search for ‘evolution-proof' antibiotics. Trends Microbiol.

[5] Schulz zur Wiesch P, Engelstadter J, Bonhoeffer S (2010). Compensation of fitness costs and reversibility of antibiotic resistance mutations. Antimicrob Agents Chemother.

[6] Kluytmans J. Plasmid-encoded colistin resistance: mcr-one, two, three and counting. Euro Surveill. 2017;22 pii: 30588. 10.2807/1560-7917.ES.2017.22.31.30588 PMC5553061 28797321

[7] Yin W, Li H, Shen Y, Liu Z, Wang S, Shen Z, et al. Novel plasmid-mediated colistin resistance gene mcr-3 in Escherichia coli. MBio. 2017;8 pii: e00543–17. 10.1128/mBio.00543-17 PMC5487729 28655818

[8] Yang Q, Li M, Spiller OB, Andrey DO, Hinchliffe P, Li H (2017). Balancing mcr-1 expression and bacterial survival is a delicate equilibrium between essential cellular defence mechanisms. Nat Commun.

[9] Carrique-Mas JJ, Trung NV, Hoa NT, Mai HH, Thanh TH, Campbell JI (2015). Antimicrobial usage in chicken production in the Mekong Delta of Vietnam. Zoonoses Public Health.

[10] EMA. EMA updates its advice on the use of colistin in animals. Vet Rec. 2016;179:131–2.

[11] MacLean RC, San Millan A (2019). The evolution of antibiotic resistance. Science.

[12] Hughes D, Andersson DI (2015). Evolutionary consequences of drug resistance: shared principles across diverse targets and organisms. Nat Rev Genet.

