# Supplementary material for: Compensatory mutations modulate the competitiveness and dynamics of plasmid-mediated colistin resistance in Escherichia coli clones
Source: ISME J. 2020 Jan 2;14(3):861–5. doi: 10.1038/s41396-019-0578-6 (PMC7031280; doi:10.1038/s41396-019-0578-6)
Supplement: Supplementary file 1 — Supplemental Material [file 41396_2019_578_MOESM1_ESM.docx]

**Compensatory mutations modulate the competitiveness and dynamics of plasmid-mediated colistin resistance in *Escherichia coli* clones**

Qiu E Yang^1*^, Craig MacLean^2^, Andrei Papkou^2^, Manon Pritchard^3^, Lydia Powell^3^, David Thomas^3^, Diego O. Andrey^1,4^, Mei Li^1^, Brad Spiller^1^, Wang Yang^5^, Timothy R. Walsh^1*^

**This PDF file includes:**

- Materials and methods
- Supplementary Figures 1 and 2
- Supplementary Tables 1 to 16

**Materials and Methods**

**Studied strains and plasmid constructs**

The details of studied plasmids and strains were listed in **supplementary Table 1**. The *mcr-3.5* coding region was PCR amplified from strains PN218 using primers *mcr-3.5*-F and *mcr-3.5*-R (**supplementary** **Table 2**). The fragment was purified by gel purification kit (Qiagen, Germany), digested with *Pst1* and *EcoRI* enzymes (NEB, UK), and cloned into pUC19 and pBAD vectors. The resulting plasmids were confirmed by PCR using the primers (*mcr-3.1-*F and *mcr-3.1-*R, in **supplementary Table 2**) and the following cycling conditions: 30 cycles of 95°C for 30 s, 50°C for 30 s, and 72°C for 45 s, followed by 1 cycle of 72 °C for 7 min. The presumptive 542-bp PCR product of *mcr-3* was sent for sequencing (Eurofins Genomics, Germany).

**Measurement of bacterial growth rates**

Overnight cultures of three *mcr-1-, mcr-3.1- and mcr-3.5-* recombinant strains, namely, *E. coli* TOP10 (*mcr-3.1*/pBAD), *E. coli* TOP10 (*mcr-3.5*/pBAD), *E. coli* TOP10 (pBAD only) and *E. coli* TOP10], were diluted into fresh Lysogeny broth (LB) (ThermoFisher, U.K.) supplemented with 100 mg/L ampicillin (Fisher Chemical, UK) at 37°C (220 r.p.m.). Bacterial optical density was measured in every one hour using a microplate reader (EZ Read 400, BIOCHROM, U.K.) at 492nm, when cell density reached to OD_492_=0.4, L-arabinose (0.2%, w/v) was added to induce the expression of *mcr-* genes.  Bacteria with no L-arabinose induction was serviced as controls and three biological repeats were conducted in this experiment.

**Dead-live assay by florescent microscopy**

*E. coli* TOP10 (*mcr-3.1*/pBAD), *E. coli* TOP10 (*mcr-3.5*/pBAD), *E. coli* TOP10 (pBAD only) and *E. coli* TOP10 (n=4 cultures/strain) were grown overnight in LB broth supplemented with 100 mg/L ampicillin (Fisher Chemical, UK) at 37°C (220 r.p.m.).  Overnight cultures were standardized to OD_600_ 0.05 and inoculated (1:10; v/v) into 96-well glass-bottomed plates (Whatman^®^, UK) in LB broth for 16 h (37°C; 30 r.p.m.).  The supernatant was gently removed and the biofilms were further incubated in fresh LB broth ± L-arabinose (0.2%, w/v) for 8 h.  The supernatant was removed and the biofilms stained with 6% LIVE/DEAD^®^ (v/v; BacLight^TM^ Bacterial Viability Kit, Invitrogen) in phosphate buffered saline (PBS) prior to confocal laser scanning microscopy (CLSM) imaging (Leica TCS SP5) with ax63 lens. The CLSM z-stack images were analysed using COMSTAT image analysis software for quantification of biofilm biomass. Statistical software (Minitab v.14; Minitab, State College, PA) was used for statistical analyses presented. The non-parametric data was analysed using Mann-Whitney test to determine significant differences for pair-wise comparisons.

**Morphological analysis by transmission electron microscopy (TEM)**

Overnight cultures were diluted into 50 ml of fresh LB broth supplemented with 100 mg/L ampicillin for *mcr-3*/pBAD. 0.2% (w/v) of L-arabinose was added to induce the overexpression of *mcr-3* and after 8h incubation, samples were fixed by addition of glutaraldehyde to the broth to a final concentration of 1%, as previously described [1]. In brief, bacteria were harvested by collection onto 0.45 mm pore filters, gently scraped off and dispersed in 4% low melting point agarose at 50^o^C. The gel was then cut into 1 mm cubes, which followed by post-fixing for 2 h in 2% uranyl acetate. Once fixed, cubes were washed in reverse osmosis purified water three times (20 min per time), and dehydrated using graded propan-2-ol (10 min each for 50%, 70%, 90%, and 2x 15 min for 100%). Before thin section (80nm), the cubes were infiltraded with LR White acrylic resin (London Resin Company, Aldermaston, U. K.) and then placed in size 0 gelatine capsules with fresh resin and heat polymerised overnight at 50^o^C. AnalySIS (Soft Imaging System GmbH, Germany) and a Megaview III digital camera were used to observe cell wall structures.

**Generation of site-directed point mutations in *mcr-3*-encoding sequence region**

A total of six targeted mutations were generated at 1626bp *mcr-3.5* sequence using the Q5® Site-Directed mutagenesis kit (NEB, U.K). Primers were designed by NEBaseChanger (<http://nebasechanger.neb.com/>), as specified in **supplementary** **Table 3**. In brief, PCR amplicons were acquired using specific primers and a master mix containing Q5 hot start High-Fidelity DNA polymerase (Invitrogen, U.K), follows by incubated with kinase-ligase-DphI (KLD) enzyme mix. This step is designed for the circulation of the PCR product and removal of the template. Then the ligated plasmid DNA was transferred into chemically competent TOP10 cells (ThermoFisher, UK). The mutations were confirmed by plasmid extraction and sequencing.

**Bacterial competitiveness measured by flow cytometry**

*In-vitro* competition experiments were used to measure the relative fitness of the *mcr-3.1/*pBAD, *E. coli* TOP10 (*mcr-3.5*/pBAD) and *E. coli* TOP10 (pBAD only) and *E. coli* TOP10. These strains were competed against a GFP-labelled *E. coli* DH5-α carrying plasmid pHT315-pAphA3’-gfp for constitutive expression [2]. All competitions were carried out in M9 medium (SIGMA-ALDRICH, U.K) with six biological replicates per strain/condition, as previously described with some modifications [1,3]. In brief, studied strains were incubated at 37^o^C with 220 rpm shaking overnight in 5 ml of LB broth. The overnight cultures were diluted 1:400 in M9 broth and mixed at a ratio of 50% studied strains and 50% GFP-labelled control strains*.* The exact initial proportions were confirmed via flow cytometry using an Accuri C6 flow cytometer (Becton Dickenson, Biosciences, U.K). The expression of *mcr*-like genes was induced by adding 0.2% (w/v) of L-arabinose at zero time point. Performing a competition between a tested strain and a GFP-tagged competitor strain, a pure culture of each as controls are needed. Mixtures were diluted 400-fold in fresh LB broth supplemented with 0.2% arabinose and competed for 10 h at 37^o^C with 220 rpm shaking. After 10 h incubation, the competed bacteria were diluted 1:400 in Nunclon^TM^ Delta Surface 96-well plates (Therom Scientific, UK) with M9 medium and analyzed on a flow cytometer Accuri C6. For each competition, we ensured that the GFP-labelled strain can be well separated from non-fluorescent strains by comparing non-mixed controls (overlap is usually less than 2% of the cells). Formula used for the calculation of relative fitness was described in previous study [1].

**Analysis of fitness epistasis and statistical significance:** A multiplicative model was used to calculate the expected fitness, W*_AB(expected)_=* W*_A_* x W*_B_*, Where W*_AB_* is the fitness of strains carrying mutated alleles *A* and *B*. The epistasis interactions (*ε*) between mutations is estimated by the following equation: *ε* = *W_AB(observed)_ –* *W_AB(expected)_*, where the error (σ) of the *ε* is then calculated using the method in previously described [4].

**The dynamics of both *mcr-1-* and *mcr-3-* plasmids by real-time quantitative PCR (qPCR)**

**(1) *mcr-*carrying plasmids competition in *E. coli* J53 model**

To examine the stability and competitive ability of *mcr-1*- and *mcr-3*- carrying plasmids, the qPCR was used to measure changes in the abundance of *mcr-* genes over the course of serial passage (14 days). Two representative *mcr-1* (PN23, IncX4) and *mcr-3* (F203, IncF) plasmids were transferred into the same recipient genetic background (*E. coli* J53) by conjugation, generating transconjugants J53::*mcr-1* and J53::*mcr-3*, respectively (supplementary Table S1). Each competition was initiated with 1:1 mixture (with equal bacterial colony-FORMING unit per mililiter, CFU/ml) of *mcr-1* and *mcr-3.5* plasmid bearing bacteria in 5 ml tubes containing 3 ml of LB broth. Competitions were maintained for at least 80 generations of growth by serially transferring 1% of the overnight mixtures to 3 ml fresh medium with or without colistin (2 mg/l) for 14 days. The negative controls, *mcr-1* or *mcr-3*-plasmid monocultures, were also processed in the same manner. Biological triplicates were performed in each treatment.

**(2) *mcr*-carrying Plasmids competition in a clinical strain model**

Co-existence of *mcr-1* and *mcr-3* plasmids were found in our MCRPEC collection, in this model, three MCRPEC strains co-existed *mcr-1* and *mcr-3.5* (**supplementary Table 1**) were selected and examined the stability and competitiveness of both *mcr-1*- and *mcr-3*-carrying plasmids in the presence/lackness of colistin, the qPCR was used to measure changes in plasmid abundance over 14-day passage as above describe with slightly differences. Briefly, each competition was initiated with equal CFU/ml in 5 ml tubes containing 3 ml of LB broth. Competitions were maintained for at least 100 generations of growth by serially 1:1000 dilution of the overnight mixtures to fresh LB medium supplemented with (4 mg/l, as MICs of three studied strains are 8 ml/l) or without colistin for 14-day serial passaging. Biological triplicates were performed in each strain per treatment.

**(3) Plasmid abundance measured by qPCR**

In preparation of qPCR, cell pellets were collected after centrifugation (5000 rpm for 10 min) of 2 ml per overnight culture at six different time points (day0, 1, 3, 5, 8, 11 and day 14), followed by total genomic DNA (gDNA) extraction using a QIAcube automated machine (Qiagen, Germany). The concentrations of purified gDNA were measured by a Qubit (ThermoFisher Scientific, UK). All qPCR reactions were performed in a StepOnePlus^TM^ qPCR machine (Applied Biosystems, U.K.) with following conditions: 95^o^C for 10 min, 40 cycles of 95^o^C for 15 seconds and 60^o^C for 30 seconds. 1 ng of gDNA was used as template with specific *mcr-1* and *mcr-3-* primers and probes, and a housekeeping gene *rpoB* was used as internal control using primers *rpoB-qF*, *rpoB-qR* and *rpoB* probe (supplementary **Table 2**), together with Precision 2xqPCR Mastermix (PrimerDesign, U.K) following manufactures’ protocol.

**(4) Statistical analysis of qPCR data**

We used threshold cycle values (*C_T_*) measured by qPCR in order to estimate relative copy number. Assuming similar and high efficiencies of all qPCR reactions, relative copy number can be expressed as 2^(-Δ^*^CT^*^)^, where Δ*C_T_* is a difference in *C_T_* for two genes within the same sample. We estimated *mcr-1* and *mcr-3* abundance relative to chromosomally encoded gene *rpoB* (1-2) and *mcr-3* copy number relative to *mcr-1* (3):

1. Δ*C_T_* (*mcr-3/rpoB*) *= C_T_* (*mcr-3*) - *C_T_* (*rpoB*)
2. Δ*C_T_* (*mcr-1/rpoB*) *= C_T_* (*mcr-1*) *- C_T_* (*rpoB*)
3. Δ*C_T_* (*mcr-3/mcr-1*) *= C_T_* (*mcr-3*) *- C_T_* (*mcr-1*)

To compare plasmid copy number across time and in different treatments, we performed regression analysis. Δ*C_T_* estimates were used directly as response variable. Time expressed as a number of generations was used as a covariate. (We estimated log_2_(1000) ≈ 10 bacterial generations per passage based on 1000-fold dilution and the fact that bacterial cultures were grown until saturation each transfer). Because the copy number changed over time in a non-linear manner (i.e. we observed both increase and decrease), we used polynomial regression with 3 orthogonal polynomial coefficients. Model diagnostics revealed that the model provided a good fit, and 2^nd^ and 3^rd^ degree polynomial coefficients were highly significant suggesting that this model is appropriate for our data.

To incorporate the effects of colistin, host strain or gene (*mcr-1* or *mcr-3*), the corresponding fixed factors and their interactions were included to the model. In addition, to account for the non-independence arising because the same cultures were measured repeatedly during the experiments, we included replicated identity as a random effect (fitting random intercepts and slopes). We fitted separate models for competitions performed in clinical isolated and in J53 strain, as well as for Δ*C_T_* relative to *rpoB* and for Δ*C_T_* (*mcr-3/mcr-1*). The full models were further used to test specific hypothesis in post-hoc comparisons. The resulting *p*-values were adjusted for multiple comparisons following Holm-Bonferroni procedure. The regression analysis was carried out in R version 3.5.1 [5]. The mixed model was fitted using package lme4 (version 1.1-17) [6], *F*-statistics and *p*-values were obtained using lmerTest (version 3.0-1) [7], and *post-hoc* comparisons were performed using package emmeans (version 1.3.0) [8].

**Supplementary Figures**


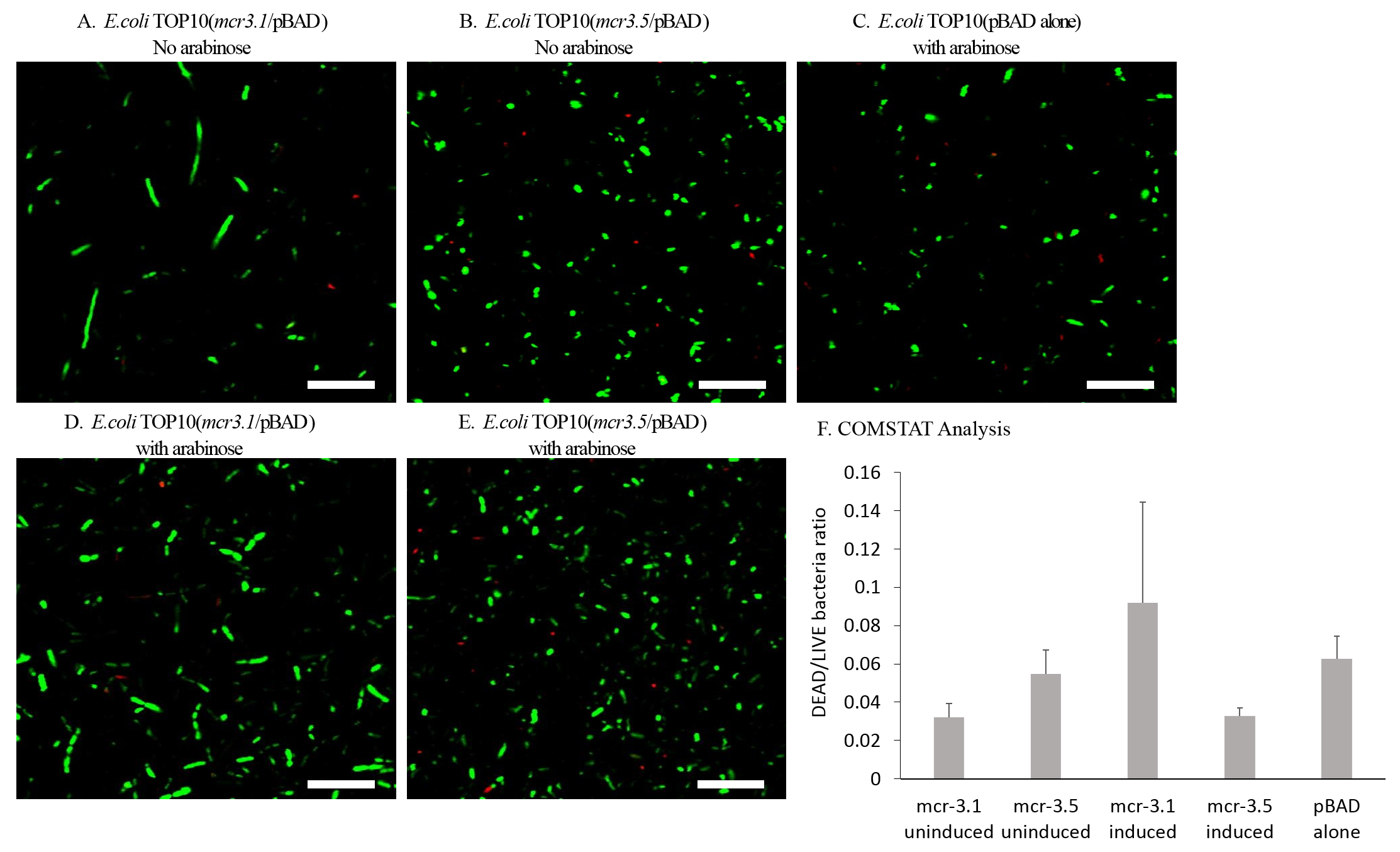


**Supplementary Fig.1** The toxic effects of *mcr-3* overexpression on cell viability. **A-D**, Representative Confocal Laser Scanning microscopy images of cells treated with/without L-arabinose and stained with LIVE/DEAD^®^. Live and dead cells presented green and red colour, respectively. Scale bar is 15 µm. **D**, Ratio of Dead to Live bacteria (biomass) obtained from CLSM z-stack images through COMSTAT analysis of *E. coli* biofilms grown for 16 h in LB broth, followed by ± L-arabinose (0.2% w/v; 8 h) treatment, where the biofilms were stained with LIVE/DEAD^®^ (n=4; error bars show standard error). Statistical software (Minitab v.14; Minitab, State College, PA) was used for statistical analyses presented. The non-parametric data was analyzed using Mann-Whitney test to determine significant differences for pair-wise comparisons (supplementary Table 4).


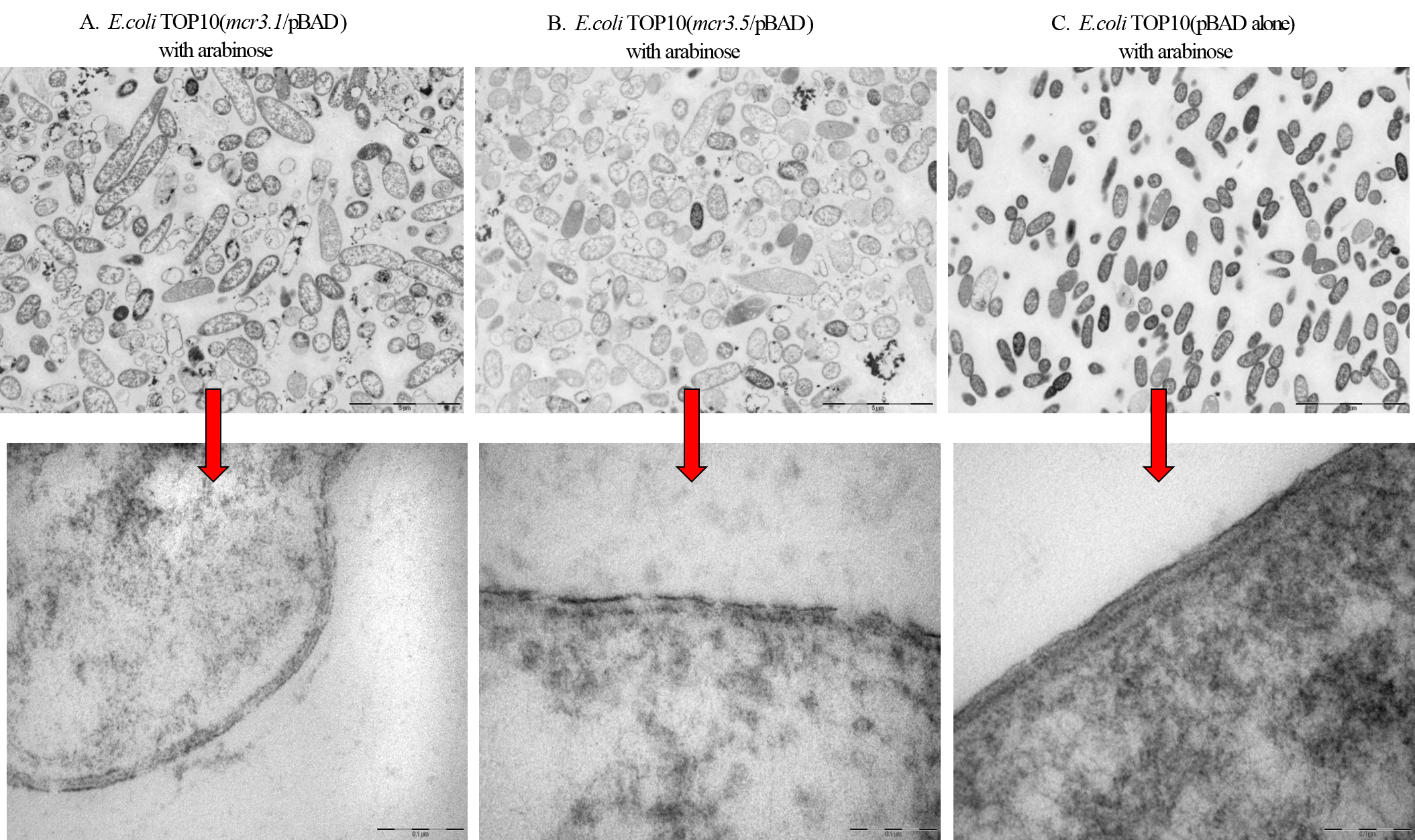


**Supplementary Fig.2** TEM micrographs of *mcr-3.1* and *mcr-3.5* over-producing cells in A and B, respectively, the damaged outer-membrane and some completely lysed cells were observed. C. TEM micrographs of of control strain TOP10 (pBAD alone) *E. coli*, exhibited the intact outer membrane structure and highly homologous electron density.

**Supplementary Tables**

**Supplementary Table 1** the detail of stains and plasmids studied in this work

| Strains ID | Source | Containing *mcr-* genes | Applications | Reference |
| --- | --- | --- | --- | --- |
| pABD | Invitrogen, U.K. | - | Expression vector | (1) |
| *mcr-*1/pABD | - | - | Fitness study | (1) |
| *mcr-*3.1/pABD | - | - | Fitness study | This study |
| *mcr-*3.5/pABD | - | - | Fitness study | This study |
| *E. coli* J53 | - | - | recipient in conjugation assay | - |
| *E. coli* TOP10 | Invitrogen, U.K. | - | transformation | - |
| PN42 | human | *mcr-1, mcr-3.1*  (co-existence) | competition models | (1) |
| PN4 | water | *mcr-1, mcr-3.1*  (co-existence) | competition models | This study |
| PN24 | duck feces | *mcr-1, mcr-3.1*  (co-existence) | competition models | (1) |
| *E. coli* J53::*mcr-1** | *mcr-1* plasmid obtained from duck feces | *mcr-1* | competition models  （1:1 mixed with *E. coli* J53::*mcr-3*） | This study |
| *E. coli* J53::*mcr-3.5** | *mcr-3.5* plasmid obtained from blowflies | *mcr-3.5* | competition models  （1:1 mixed with *E. coli* J53::*mcr-1*） | This study |

‘-’ Indicates not applicable. ‘*’ represents transconjugants

**Supplementary Table 2** primers of gene *mcr-1* and *mcr-3* for construction, screening and qPCR

| primer name | Sequence (5'-3') | length | Tm (°C) | application | reference |
| --- | --- | --- | --- | --- | --- |
| *mcr-1*-F | GCTACTGATCACCACGCTGT | 958bp | 60 | PCR screening | (1) |
| *mcr-1*-R | TGGCAGCGACAAAGTCATCT |  |  |  |  |
| *mcr-3.1*-F | TTGGCACTGTATTTTGCATTT | 542bp | 50 | PCR screening | (2) |
| *mcr-3.1*-R | TTAACGAAATTGGCTGGAACA |  |  |  |  |
| *mcr-3.5*-F-*pstI* | AAAACTGCAGATGTTACAATGTGGGAGTATCAG | 1863bp | 60 | plasmid construct | This study |
| *mcr-3.5*-R-  *EcoRI* | CGGAATTCCAGATGATTGGGGGCCTGA |  |  |  |  |
| *rpoB*-qF | TCCTTTCTATCCAGCTTGACTCGT | ~200bp | 60 | qPCR | (1) |
| *rpoB*-qR | CGCAGTTTAACGCGCAGCGG |  |  |  |  |
| *rpoB* Ec probe | HEX-ACGTCAGCTACCGCCTTGGCGAACCGGTGT-BHQ1 |  |  |  |  |
| *mcr-3*-qF | CGTGTTCCTATGCAGGTGTG | ~150bp | 60 | qPCR | This study |
| *mcr-3*-qR | CGAGTATCAGCGGCTTTCTG |  |  |  |  |
| *mcr-3*-probe | FAM-TGCAAACACGCCATATCAACGCCT-BHQ1 |  |  |  |  |
| *mcr-1-*qF | TGGCGTTCAGCAGTCATTAT | ~120bp | 60 | qPCR | (1) |
| *mcr-1*-qR | AGCTTACCCACCGAGTAGAT |  |  |  |  |
| *mcr-1*-probe | FAM-AGTTTCTTTCGCGTGCATAAGCCG-BHQ1 |  |  |  |  |

**Supplementary Table 3** primers for site-direct mutations in *mcr-3.1*-encoding sequencing region

| Plasmid template | amino acid substitution (nucleotide mismatch) | primers | Resultant mutation (amino acid substitution) |
| --- | --- | --- | --- |
| mcr-3.5/pBAD | M23V+A457E+T488I | See supplementary **Table 2** | *mcr-3.5* |
| mcr-3.1/pBAD | M23V(a67g)_forward | TTTTGCATTTaTGCTGAACTG | *mcr-3* (E457A+ I488T) |
|  | _reverse | TACAGTGCCAAAAAGAAC |  |
| mcr-3.5/pBAD | A457E (c1370a) _forward | TCACTGGGAGcATTAGGGCTTTAC | *mcr-3* (M23V+ T488I) |
|  | _reverse | TTCACCATGATCGGAGAC |  |
| mcr-3.5/pBAD | I488T(c1463t) _forward | CCTGGATTTAcCAAAGAGAAAGGC | *mcr-3*(V23M+ E457A) |
|  | _reverse | TGACATCCACACCTGCAT |  |
| *mcr-3* (M23V+ T488I) | I488T(c1463t) | Primers see above | *mcr-3* (M23V) |
| *mcr-3* (M23V+ A457E | M23V(a67g) | Primers see above | *mcr-3* (A457E) |
| *mcr-3* (A457E+ T488I) | A457E (c1370a) | Primers see above | *mcr-3* (T488I) |

* *mcr-3.1*/pBAD plasmid was provided by our collaborators in China. mismatched nucleotides are indicated in red.

**Supplementary Table 4** pair-wise significance comparisons for the analysis of dead/alive data

| Sample 1 | Sample 2 | *P* value* |
| --- | --- | --- |
| *mcr-3.1* uninduced | *mcr-3.5* uninduced | 0.0000 |
| *mcr-3.1* uninduced | *mcr-3.1* Induced | 0.0001 |
| *mcr-3.5* uninduced | *mcr-3.5* Induced | 0.0000 |
| *mcr-3.1* Induced | *mcr-3.5* Induced | 0.0002 |
| *mcr-3.1* uninduced | pBAD alone | 0.0000 |
| *mcr-3.5* Induced | pBAD alone | 0.0000 |

******p* value calculation: Statistical software (Minitab v.14; Minitab, State College, PA) was used for statistical analyses presented. The non-parametric data was analysed using Mann-Whitney test to determine significant differences for pair-wise comparisons, *p*<0.05 was considered statistically significant. All other comparisons were not significant.

**Supplementary Table 5** Relative Fitness values of MCR-3 mutants *

*This table shows the fitness values of mutants that were used to reconstruct the MCR-3 fitness landscape. We measured the fitness of each mutant in direct competition with GFP marked reference strain *E. coli* DH5-α, and fitness values were standardized relative to the mean fitness of MCR-3.1. Two outliers with radically different fitness values (>40% different to median estimate for mutant) were excluded from analysis.

**Supplementary Table 6** Significance tests of fitness data*

*We compared the fitness of mutant and parent strains in the MCR fitness landscape using 2-tailed *t*-tests, assuming constant variance between samples. Highlighted *P* values show statistically significant fitness differences, after Bonferonni correcting for multiple testing (P_crit_=.05/12).

**Supplementary Table 7** Epistasis interactions between three amino acid substitutions in MCR-3.1.

| Genotype | Observed fitness | Expected fitness | Epistatic interaction term (ε) | Error in epistatic interaction term (σ(ε)) |
| --- | --- | --- | --- | --- |
| M23V+A457V | 1.299 | 1.447 | -0.148 | .098 |
| M23V+T488I | 1.433 | 1.482 | -0.049 | .120 |
| A457V+T488I | 1.449 | 2.052 | -0.603 | .106 |
| MCR-3.5  (M23V+A457V+T488I) | 1.292 | 2.098 | -0.806 |  |

*This table shows epistatic interactions between substitutions in MCR3. Expected fitness was calculated according to a multiplicative model of fitness (i.e., expected W_A+B_= W_A_* W_B_) and we calculated epistatic interaction terms as the difference between observed and expected fitness. We calculated the error in pairwise epistatic interaction terms using methods in Trindade et al. (3), and we consider epistasis to be significant when |**ε|>σ(ε).**

**Supplementary Table 8**. Nonlinearity of change in *mcr-1* and *mcr-3* copy number in clinical strains across time as indicated by polynomial regression^^[[1]](#footnote-1)^*^

|  | *Response variable:* *–*Δ*Ct* (*mcr*/*rpoB*) | | | |  |
| --- | --- | --- | --- | --- | --- |
| Term | Estimate | Std. Error | *t* statistics | | *p*-value |
|  |  |  |  | |  |
| generation, poly 1 | -2.895^**^ | 0.898 | -3.224 | | 0.00147 |
| generation, poly 2 | 8.090^***^ | 0.898 | 9.010 | | <0.00001 |
| generation, poly 3 | 2.448^**^ | 0.898 | 2.726 | | 0.00697 |
| Constant | -1.211^***^ | 0.062 | -19.496 | | <0.00001 |
|  | |  |  | |  |
| Observations | 209 | |  | |  |
| R^2^ | 0.326 | |  | |  |
| Adjusted R^2^ | 0.316 | |  | |  |
| Residual Std. Error | 0.898 (df = 205) | |  | |  |
| F Statistic | 33.002^***^ (df = 3; *N*=205; p <0.00001) | |  | |  |
|  | |  |  | |  |
| *Note:* | ^*^ p<0.05 ^**^p<0.01 ^***^ p<0.001 | | |  | |

**Supplementary Table 9**. Regression analysis of change in *mcr-1* and *mcr-3* copy number relative to *rpoB* in wild-type strains^^[[2]](#footnote-2)^*^

| **Term** | **Sum Sq** | **Mean Sq** | **NumDF** | **DenDF^^[[3]](#footnote-3)^^**^*^ | **F value** | **Pr(>F)** |
| --- | --- | --- | --- | --- | --- | --- |
| *Response variable:* *–*Δ*Ct* (*mcr*/*rpoB*) | | | | | | |
| Generation (poly) | 75.85 | 25.28 | 3 | 51.20 | 68.87 | **<0.0001** |
| Gene | 20.42 | 20.42 | 1 | 24.83 | 55.62 | **<0.0001** |
| Strain | 5.57 | 2.78 | 2 | 24.80 | 7.58 | **0.0027** |
| Colistin | 0.13 | 0.13 | 1 | 24.83 | 0.34 | 0.5640 |
| Generation (poly) × Gene | 21.94 | 7.31 | 3 | 51.20 | 19.92 | **<0.0001** |
| Generation (poly) × Strain | 6.02 | 1.00 | 6 | 51.38 | 2.73 | **0.0222** |
| Gene × Strain | 2.59 | 1.29 | 2 | 24.80 | 3.53 | **0.0449** |
| Generation (poly) × Colistin | 1.90 | 0.63 | 3 | 51.20 | 1.72 | 0.1736 |
| Gene × Colistin | 0.46 | 0.46 | 1 | 24.83 | 1.26 | 0.2725 |
| Strain × Colistin | 0.70 | 0.35 | 2 | 24.80 | 0.95 | 0.3986 |
| Generation (poly) × Gene × Strain | 1.86 | 0.31 | 6 | 51.38 | 0.84 | 0.5413 |
| Generation (poly) × Gene × Colistin | 0.51 | 0.17 | 3 | 51.20 | 0.46 | 0.7113 |
| Generation (poly) × Strain × Colistin | 1.10 | 0.18 | 6 | 51.38 | 0.50 | 0.8056 |
| Gene × Strain × Colistin | 0.37 | 0.18 | 2 | 24.80 | 0.50 | 0.6124 |
| Generation (poly)×Gene×Strain ×Colistin | 2.29 | 0.38 | 6 | 51.38 | 1.04 | 0.4105 |

**Supplementary table 10**. Regression analysis of change of *mcr-3* copy number relative to *mcr-1* in wild-type strains^^[[4]](#footnote-4)^*^

| **Term** | **Sum Sq** | **Mean Sq** | **NumDF** | **DenDF^^[[5]](#footnote-5)^^**^*^ | **F value** | **Pr(>F)** |
| --- | --- | --- | --- | --- | --- | --- |
| *Response variable:* *–*Δ*Ct* (*mcr-3*/*mcr-1*) | | | | | | |
| Generation (poly) | 44.09 | 14.70 | 3.00 | 24.61 | 50.79 | **<0.0001** |
| Strain | 2.39 | 1.20 | 2.00 | 12.25 | 4.14 | **0.0424** |
| Colistin | 0.31 | 0.31 | 1.00 | 12.26 | 1.06 | 0.3228 |
| Generation (poly) × Strain | 3.80 | 0.63 | 6.00 | 24.73 | 2.19 | 0.0786 |
| Generation (poly) × Colistin | 1.01 | 0.34 | 3.00 | 24.61 | 1.16 | 0.3440 |
| Strain × Colistin | 0.62 | 0.31 | 2.00 | 12.25 | 1.08 | 0.3704 |
| Generation (poly) × Strain × Colistin | 3.99 | 0.66 | 6.00 | 24.73 | 2.30 | 0.0669 |

**Supplementary Table 11**. The estimates of *mcr-3* copy number relative to *mcr-1* in wild-type strains with no colistin^^[[6]](#footnote-6)^*^

| **Strain** | **Generation** | **Estimate** | **95% CI** | **Std. Error** | ***d.f.*** | ***t* statistics** | ***p-*value** |
| --- | --- | --- | --- | --- | --- | --- | --- |
| *Response variable:* *–*Δ*Ct* (*mcr-3*/*mcr-1*), no colistin | | | | | | | |
| PN24 | 10 | 1.428 | -0.070,2.926 | 0.412 | 22.86 | 3.467 | 0.056018 |
|  | 30 | 0.282 | -1.032,1.597 | 0.369 | 26.90 | 0.765 | 1.000000 |
|  | 50 | -0.028 | -1.250,1.194 | 0.353 | 36.53 | -0.079 | 1.000000 |
|  | 80 | 0.487 | -0.529,1.503 | 0.284 | 26.07 | 1.716 | 1.000000 |
|  | 110 | 1.374 | 0.225,2.524 | 0.326 | 29.89 | 4.215 | **0.006368** |
|  | 140 | 1.728 | 0.369,3.087 | 0.373 | 22.37 | 4.638 | **0.003793** |
| PN4 | 10 | 0.277 | -1.206,1.760 | 0.404 | 21.29 | 0.686 | 1.000000 |
|  | 30 | -0.373 | -1.604,0.858 | 0.318 | 15.67 | -1.176 | 1.000000 |
|  | 50 | -0.563 | -1.680,0.555 | 0.310 | 24.17 | -1.818 | 1.000000 |
|  | 80 | -0.080 | -1.092,0.933 | 0.282 | 25.54 | -0.282 | 1.000000 |
|  | 110 | 1.190 | 0.063,2.317 | 0.317 | 27.16 | 3.758 | **0.023237** |
|  | 140 | 3.095 | 1.738,4.453 | 0.372 | 22.16 | 8.330 | **0.000001** |
| PN42 | 10 | 1.776 | 0.293,3.260 | 0.404 | 21.29 | 4.399 | **0.007049** |
|  | 30 | 1.062 | -0.169,2.293 | 0.318 | 15.67 | 3.344 | 0.101342 |
|  | 50 | 0.635 | -0.483,1.752 | 0.310 | 24.17 | 2.051 | 1.000000 |
|  | 80 | 0.526 | -0.487,1.538 | 0.282 | 25.54 | 1.862 | 1.000000 |
|  | 110 | 1.046 | -0.081,2.173 | 0.317 | 27.16 | 3.304 | 0.067061 |
|  | 140 | 2.185 | 0.828,3.542 | 0.372 | 22.16 | 5.880 | **0.000213** |

**Supplementary Table 12**. The estimates of *mcr-3* copy number relative to *mcr-1* in clinical at the presence of colistin^^[[7]](#footnote-7)^*^

| **Strain** | **Generation** | **Estimate** | **95% CI** | **Std. Error** | ***df*** | ***t* statistics** | ***p-*value** |
| --- | --- | --- | --- | --- | --- | --- | --- |
| *Response variable:* *–*Δ*Ct* (*mcr-3*/*mcr-1*), colistin | | | | | | | |
| PN24 | 10 | 1.166 | -0.317,2.649 | 0.404 | 21.29 | 2.888 | 0.191993 |
|  | 30 | 0.342 | -0.889,1.573 | 0.318 | 15.67 | 1.078 | 1.000000 |
|  | 50 | 0.156 | -0.962,1.273 | 0.310 | 24.17 | 0.503 | 1.000000 |
|  | 80 | 0.684 | -0.328,1.697 | 0.282 | 25.54 | 2.424 | 0.477703 |
|  | 110 | 1.653 | 0.526,2.780 | 0.317 | 27.16 | 5.221 | **0.000546** |
|  | 140 | 2.465 | 1.108,3.823 | 0.372 | 22.16 | 6.634 | **0.000038** |
| PN4 | 10 | 0.481 | -1.003,1.964 | 0.404 | 21.29 | 1.191 | 1.000000 |
|  | 30 | -0.360 | -1.591,0.871 | 0.318 | 15.67 | -1.133 | 1.000000 |
|  | 50 | -0.594 | -1.712,0.523 | 0.310 | 24.17 | -1.920 | 1.000000 |
|  | 80 | -0.182 | -1.194,0.831 | 0.282 | 25.54 | -0.643 | 1.000000 |
|  | 110 | 0.639 | -0.488,1.767 | 0.317 | 27.16 | 2.019 | 1.000000 |
|  | 140 | 1.296 | -0.062,2.653 | 0.372 | 22.16 | 3.486 | 0.056018 |
| PN42 | 10 | 0.392 | -1.093,1.876 | 0.404 | 21.42 | 0.968 | 1.000000 |
|  | 30 | 0.408 | -0.826,1.642 | 0.320 | 16.17 | 1.273 | 1.000000 |
|  | 50 | 0.418 | -0.701,1.537 | 0.310 | 24.31 | 1.348 | 1.000000 |
|  | 80 | 0.552 | -0.495,1.599 | 0.296 | 29.22 | 1.863 | 1.000000 |
|  | 110 | 1.010 | -0.213,2.232 | 0.351 | 34.43 | 2.873 | 0.159205 |
|  | 140 | 1.994 | 0.635,3.352 | 0.372 | 22.25 | 5.357 | **0.000688** |

**Supplementary Table 13**. Regression analysis of changes in *mcr-1* and *mcr-3* copy number relative to *rpoB* during the competition within *E.coli* J53^^[[8]](#footnote-8)^*^

| **Term** | **Sum Sq** | **Mean Sq** | **NumDF** | **DenDF^^[[9]](#footnote-9)^^**^*^ | **F value** | **Pr(>F)** |
| --- | --- | --- | --- | --- | --- | --- |
| *Response variable:* *–*Δ*Ct* (*mcr*/*rpoB*) | | | | | | |
| Generation (poly) | 115.99 | 38.66 | 3 | 88.00 | 92.19 | **<0.0001** |
| Gene | 75.38 | 75.38 | 1 | 88.00 | 179.74 | **<0.0001** |
| Culture | 1.43 | 1.43 | 1 | 88.00 | 3.42 | 0.0680 |
| Colistin | 0.05 | 0.05 | 1 | 88.00 | 0.13 | 0.7222 |
| Generation (poly) × Gene | 8.59 | 2.86 | 3 | 88.00 | 6.83 | **0.0003** |
| Generation (poly) × Culture | 8.21 | 2.74 | 3 | 88.00 | 6.52 | **0.0005** |
| Gene × Culture | 0.26 | 0.26 | 1 | 88.00 | 0.61 | 0.4361 |
| Generation (poly) × Colistin | 2.17 | 0.72 | 3 | 88.00 | 1.72 | 0.1683 |
| Gene × Colistin | 4.26 | 4.26 | 1 | 88.00 | 10.16 | **0.0020** |
| Culture × Colistin | 3.08 | 3.08 | 1 | 88.00 | 7.35 | **0.0081** |
| Generation (poly) × Gene × Culture | 9.37 | 3.12 | 3 | 88.00 | 7.45 | **0.0002** |
| Generation (poly) × Gene × Colistin | 3.35 | 1.12 | 3 | 88.00 | 2.66 | 0.0528 |
| Generation (poly) × Culture × Colistin | 2.14 | 0.71 | 3 | 88.00 | 1.70 | 0.1730 |
| Gene × Culture × Colistin | 3.69 | 3.69 | 1 | 88.00 | 8.79 | **0.0039** |
| Generation(poly)×Gene×Culture×Colistin | 0.99 | 0.33 | 3 | 88.00 | 0.79 | 0.5036 |

**Supplementary Table 14**. Regression analysis of the change of *mcr-3* relative to *mcr-1* copy number during the competition within *E.coli* J53^^[[10]](#footnote-10)^*^

| **Term** | **Sum Sq** | **Mean Sq** | **NumDF** | **DenDF^^[[11]](#footnote-11)^^**^*^ | **F value** | **Pr(>F)** |
| --- | --- | --- | --- | --- | --- | --- |
| *Response variable:* *–*Δ*Ct* (*mcr-3*/*mcr-1*) | | | | | | |
| Generation (poly) | 7.59 | 2.53 | 3.00 | 22.00 | 5.03 | 0.0084 |
| Colistin | 0.02 | 0.02 | 1.00 | 22.00 | 0.04 | 0.8407 |
| Generation (poly) × Colistin | 3.67 | 1.22 | 3.00 | 22.00 | 2.43 | 0.0919 |

**Supplementary Table 15**. The estimates of *mcr-3* copy number relative to *mcr-1* during the competition within *E.coli* J53 at the presence or absence of colistin^^[[12]](#footnote-12)^*^

| **Colistin** | **Generation** | **Estimate** | **95% CI** | **Std. Error** | ***df*** | ***t* statistics** | ***p-*value** |
| --- | --- | --- | --- | --- | --- | --- | --- |
| *Response variable:* *–*Δ*Ct* (*mcr-3*/*mcr-1*) | | | | | | | |
| Colistin | 10 | -2.983 | -4.390,-1.575 | 0.403 | 11.04 | -7.404 | **0.000132** |
|  | 30 | -2.181 | -3.273,-1.090 | 0.321 | 12.43 | -6.803 | **0.000142** |
|  | 50 | -1.398 | -2.366,-0.429 | 0.296 | 15.39 | -4.722 | **0.001530** |
|  | 80 | -0.666 | -1.917,0.586 | 0.388 | 16.99 | -1.713 | 0.104825 |
|  | 110 | -1.029 | -2.566,0.508 | 0.408 | 8.44 | -2.520 | 0.068768 |
| No colistin | 10 | -1.930 | -3.337,-0.523 | 0.403 | 11.04 | -4.790 | **0.002780** |
|  | 30 | -1.815 | -2.906,-0.724 | 0.321 | 12.43 | -5.660 | **0.000738** |
|  | 50 | -1.550 | -2.519,-0.582 | 0.296 | 15.39 | -5.237 | **0.000738** |
|  | 80 | -1.320 | -2.572,-0.068 | 0.388 | 16.99 | -3.398 | **0.010271** |
|  | 110 | -1.905 | -3.442,-0.368 | 0.408 | 8.44 | -4.664 | **0.005592** |

**Reference**

1. Yang Q, Li M, Spiller OB, Andrey DO, Hinchliffe P, Li H, MacLean C, Niumsup P, Powell L, Pritchard M, Papkou A, Shen Y, Portal E, Sands K, Spencer J, Tansawai U, Thomas D, Wang S, Wang Y, Shen J, Walsh T. 2017. Balancing mcr-1 expression and bacterial survival is a delicate equilibrium between essential cellular defence mechanisms. Nat Commun 8:2054.
2. Daou N, Buisson C, Gohar M, Vidic J, Bierne H, Kallassy M *et al* (2009). IlsA, a unique surface protein of Bacillus cereus required for iron acquisition from heme, hemoglobin and ferritin. *PLoS pathogens* **5:** e1000675.
3. San Millan A, Escudero JA, Gifford DR, Mazel D, MacLean RC (2016). Multicopy plasmids potentiate the evolution of antibiotic resistance in bacteria. *Nature Ecology & Evolution* **1:** 0010.
4. Trindade S, Sousa A, Xavier KB, Dionisio F, Ferreira MG, Gordo I (2009). Positive Epistasis Drives the Acquisition of Multidrug Resistance. *PLoS genetics* **5.**
5. Team RC (2018). R: A language and environment for statistical computing. *R Foundation for Statistical Computing, Vienna, Austria.*
6. Douglas Bates MM, Ben Bolker, Steve Walker (2015). Fitting Linear Mixed-Effects Models Using lme4. *Journal of Statistical Software* **67(1):** 1-48.
7. Kuznetsova A BP, Christensen RHB (2017). lmerTest Package: Tests in Linear Mixed Effects Models. Journal of Statistical Software 82 (13): 1-26.
8. Lenth R (2018). emmeans: Estimated Marginal Means (Least-Squares Means). R package version 130.

1. ^*^ – To model the change in *mcr-1* or *mcr-3* copy number across time we used polynomial regression. The difference in threshold cycle (Δ*Ct*) between either *mcr-1* or *mcr-3* and chromosomally encoded gene *rpoB* were used as a response variable. The highly significant coefficients for the second and third degree polynomials indicate a non-linearity in the change of response variable over time. For a full model incorporating the effect of host strain, particular gene and presence of colistin, see Supplementary Table 10. The analysis was performed using R (version 3.5.1). [↑](#footnote-ref-1)
2. ^*^ – The difference in threshold cycle (Δ*Ct*) between either *mcr-1* or *mcr-3* and chromosomally encoded gene *rpoB* were used as a response variable. The response variable changed over time in a non-linear manner (see Supplementary Table 9), therefore, we fitted a polynomial regression using 3 degree orthogonal polynomials. In addition, three fixed variables and their interactions were used as predictors: Gene (*mcr-1* or *mcr-3*), Strain (PN42, PN4 or PN24) and Colistin (colistin presense/absence). Each combination of factors Gene/Strain/Colistin included three biological replicates which were measured repeatedly over the course of the experiment. To account for non-independence among the repeated measurements, we included a replicate identity as a random effect by fitting random intercepts and slopes. The analysis was performed using R (version 3.5.1) and packages lme4 (version 1.1-17) and lmerTest (version 3.0-1). [↑](#footnote-ref-2)
3. ^*^ – Denominator degrees of freedom for *F*-statistics were calculated using R package lmerTest (version 3.0-1) using Satterthwaite's method. [↑](#footnote-ref-3)
4. ^*^ – The difference in threshold cycle (Δ*Ct*) between *mcr-3* or *mcr-1,* which is equivalent to log­­_2_ of relative copy number, were used as a response variable. The response variable was fitted against time covariate using a polynomial regression (with 3 degree orthogonal polynomials). In addition, two fixed variables and their interactions were used as predictors: Strain (PN42, PN4 or PN24) and Colistin (colistin presense/absence). Each combination of factors included three independent replicates which were measured repeatedly over the course of the experiment. To account for non-independence among the repeated measurements, we included a replicate identity as a random effect by fitting random intercepts and slopes. The analysis was performed using R (version 3.5.1) and packages lme4 (version 1.1-17) and lmerTest (version 3.0-1). [↑](#footnote-ref-4)
5. ^*^ – Denominator degrees of freedom for *F*-statistics were calculated using R package lmerTest (version 3.0-1) using Satterthwaite's method. [↑](#footnote-ref-5)
6. ^*^ – The table reports the relative copy number and associated uncertainty obtained using polynomial regression presented in Supplementary Table 11. The p-values < 0.05 indicate the estimates which differ significantly from 0, i. e. that *mcr-3/mcr-1* relative copy number differ significantly from 1. The reported *p*-values and 95% confidence intervals were adjusted with Holm-Bonferroni method (n=36) using R package emmeans (version 1.3.0). [↑](#footnote-ref-6)
7. ^*^ – The table reports the relative copy number and associated uncertainty obtained using polynomial regression presented in Supplementary Table X3. The p-values < 0.05 indicate the estimates which differ significantly from 0, i. e. that mcr-3/mcr-1 relative copy number differ significantly from 1. The reported *p*-values and 95% confidence intervals were adjusted with Holm-Bonferroni method (n=36) using R package emmeans (version 1.3.0) [↑](#footnote-ref-7)
8. ^*^ – We used Δ*Ct* between either *mcr-1* and *rpoB* or *mcr-3* and *rpoB* as a proxy for genes copy numbers. We fitted a polynomial regression (with 3 degree orthogonal polynomials of time) because the response variable showed non-linear patterns during the experiments. The polynomial coefficients were significant in the model. In addition, three fixed variables and their interactions were used as predictors: Gene (*mcr-1* or *mcr-3*), Culture (competition versus monoculture controls) and Colistin (colistin presense/absence). Each combination of factors Gene/Culture/Colistin included three biological replicates which were measured repeatedly at different time points. To account for non-independence due to the repeated measurements, we included a replicate identity as a random effect by fitting random intercepts and slopes. The analysis was performed using R (version 3.5.1) and packages lme4 (version 1.1-17) and lmerTest (version 3.0-1). [↑](#footnote-ref-8)
9. ^*^ – Denominator degrees of freedom for *F*-statistics were calculated using R package lmerTest (version 3.0-1) using Satterthwaite's method. [↑](#footnote-ref-9)
10. ^*^ – The difference in threshold cycle (Δ*Ct*) between *mcr-3* or *mcr-1,* which is equivalent to log­­_2_ of relative copy number, were used as a response variable. The response variable was fitted against time covariate using a polynomial regression (with 3 degree orthogonal polynomials). In addition, presense or absence of colistin were used as a predictor. A replicate identity was used as a random factor (fitting random intercepts and slopes). The analysis was performed using R (version 3.5.1) and packages lme4 (version 1.1-17) and lmerTest (version 3.0-1). [↑](#footnote-ref-10)
11. ^*^ – Denominator degrees of freedom for *F*-statistics were calculated using R package lmerTest (version 3.0-1) using Satterthwaite's method. [↑](#footnote-ref-11)
12. ^*^ – The table reports the relative copy number and the associated uncertainty estimated using polynomial regression (Supplementary Table 15). The *p*-values < 0.05 indicate the estimates which differ significantly from 0, i. e. that the relative copy number *mcr-3*/*mcr-1* differ significantly from 1. The reported *p*-values and 95% confidence intervals were adjusted by Holm-Bonferroni method (n=10) using R package emmeans (version 1.3.0) [↑](#footnote-ref-12)
